# Understanding the Role of Endothelial Glycocalyx in Mechanotransduction via Computational Simulation: A Mini Review

**DOI:** 10.3389/fcell.2021.732815

**Published:** 2021-08-17

**Authors:** Xi Zhuo Jiang, Kai H. Luo, Yiannis Ventikos

**Affiliations:** ^1^School of Mechanical Engineering and Automation, Northeastern University, Shenyang, China; ^2^Department of Mechanical Engineering, University College London, London, United Kingdom

**Keywords:** microscale, mesoscale, macroscale, computational simulation, mechanotransduction, endothelial glycocalyx, numerical modelling

## Abstract

Endothelial glycocalyx (EG) is a forest-like structure, covering the lumen side of blood vessel walls. EG is exposed to the mechanical forces of blood flow, mainly shear, and closely associated with vascular regulation, health, diseases, and therapies. One hallmark function of the EG is mechanotransduction, which means the EG senses the mechanical signals from the blood flow and then transmits the signals into the cells. Using numerical modelling methods or *in silico* experiments to investigate EG-related topics has gained increasing momentum in recent years, thanks to tremendous progress in supercomputing. Numerical modelling and simulation allows certain very specific or even extreme conditions to be fulfilled, which provides new insights and complements experimental observations. This mini review examines the application of numerical methods in EG-related studies, focusing on how computer simulation contributes to the understanding of EG as a mechanotransducer. The numerical methods covered in this review include macroscopic (i.e., continuum-based), mesoscopic [e.g., lattice Boltzmann method (LBM) and dissipative particle dynamics (DPD)] and microscopic [e.g., molecular dynamics (MD) and Monte Carlo (MC) methods]. Accounting for the emerging trends in artificial intelligence and the advent of exascale computing, the future of numerical simulation for EG-related problems is also contemplated.

## Introduction

The inner surface of blood vessel walls is covered by a layer of dendritic structures termed endothelial glycocalyx (EG). A typical glycocalyx unit consists of a few glycosaminoglycan (GAG) chains and a core protein which anchors the GAGs to cell membrane. The EG is exposed to the blood flow and is the first barrier in direct contact with blood. Such a unique location allows EG to coordinate microvascular mass transport (Kang et al., 2021), regulate cell adhesion (Robert et al., 2006) and participate in mechanotransduction (Tarbell and Pahakis, 2006). Mechanotransduction describes a process of bio-structures perceiving and transferring their ambient mechanical signals. Specifically for EG, mechanotransduction means the EG senses the mechanical stimuli from blood flow and transmits such signals to the cytoplasm. The response of EG to the haemodynamic forces is critical to maintaining vascular health and function (Goligorsky and Sun, 2020; Weinbaum et al., 2021). Malfunction of the EG in sensing and transmitting mechanical signals can cause severe vascular diseases such as atherosclerosis, hypertension, stroke, and sepsis (Tarbell and Cancel, 2016; Goligorsky and Sun, 2020), just to name a few.

Using wet-lab experiments, especially enzymatic degradation, is an effective way to explore the role of EG and its components in regulating mechanotransduction. Experiments have probed the glycocalyx as a mechanotrasnducer, revealed the potential manner in which the shear (mechanical stimuli) is felt and uncovered the activities involved in the message delivery after sensing the mechanical signal. Experimental evidences supporting the relationship between EG and the mechanotransduction are reviewed in Tarbell and Pahakis (2006).

Complementary to wet-lab experimental methods, *in silico* experiments, also named numerical simulations or computational modelling, approach the problems of EG mechanotransduction from a different angle. As the name itself indicates, *in silico* experiments are implemented on computers, which allow precise control of EG properties in order to delineate how EG and its components play their roles in mechanotransduction. Initially, numerical studies of EG adopted simple models only with a limited number of glycocalyx features, and the aim was mainly to reproduce wet-lab results by simulations. The recent progresses in advanced numerical methods and supercomputers empower *in silico* experiments to tackle far more challenging EG-related problems with high spatial and temporal resolutions. Breakthroughs in crystallographic structure determination lend numerical simulations the possibility to offer accurate results with a resolution at the atomic/molecular level.

The aim of this mini review is to critically summarise recent developments in numerical methods and their applications in the study of the EG mechanotransduction, demonstrating knowledge generation through *in silico* experiments. Principles of the relevant numerical methods are individually described briefly, together with their contributions to the understanding of the EG functionality as a mechanotransducer. The future of numerical studies in EG functionality is also considered, accounting for trends in artificial intelligence, big data and the advent of exascale computing.

## Numerical Methods

**FIGURE 1:**
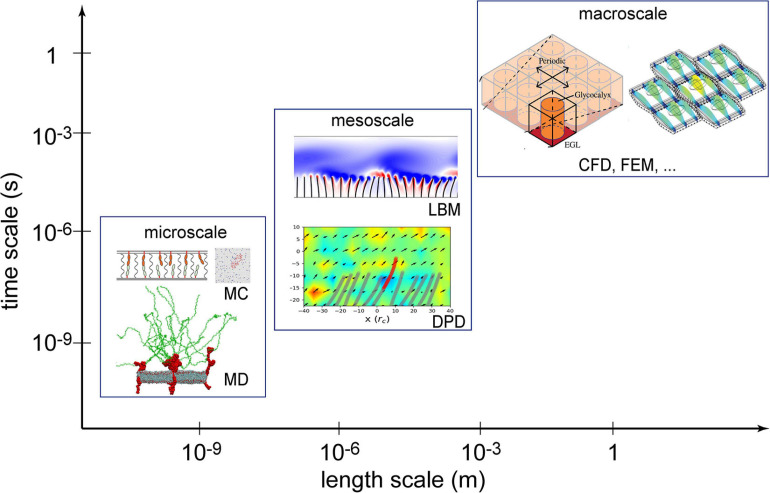
Numerical methods used in the investigation of the endothelial glycocalyx (EG) mechanotransduction. Computational fluid dynamics (CFD) and the finite element method (FEM) are two established macroscopic methods; lattice Boltzmann method (LBM) and dissipative particle dynamics (DPD) are typical mesoscopic methods; molecular dynamics (MD) and Monte Carlo (MC) are deterministic and stochastic microscopic methods, respectively. Sources: MC is from Xu et al. (2016), MD from Jiang et al. (2017), LBM from O’connor and Revell (2019), DPD from Jiang et al. (2021), CFD from Lee et al. (2020), and FEM from Dabagh et al. (2014). Reuse of figures are permitted by the Cambridge University Press, Royal Society of Chemistry and the Royal Society (United Kingdom).

Numerical methods can be classified into macroscopic, mesoscopic, and microscopic approaches in terms of length and time scales resolved, as illustrated in Figure 1. Macroscopic simulations have the longest history in solving EG-related problems and are still a popular and powerful tool. Macroscopic simulations deal with problems at cell- or tissue-scales, like endothelial cell deformation, force distribution on glycocalyx ultrastructures and flow shear stress over the EG layer (Tarbell and Shi, 2013; Dabagh et al., 2014). Mesoscopic methods can further explore detailed fluid-structure interactions (O’connor and Revell, 2019) between blood flow and endothelium surface structures but at slightly higher computational cost. Microscopic *in silico* experiments have been successfully applied to capture the atomic/molecular interactions in a single proteoglycan unit (Jiang et al., 2017). Such microscopic simulations, however, confined to a computation domain measured in nanometers and a physical time in nanoseconds, due to high computational cost.

In this section, the basic principles of these methods are described, and their applications are demonstrated. It should be noted that the main purpose of this mini review is to show advances in using numerical methods to understand the EG mechanotransduction while the algorithmic details of individual simulation methods are described in the relevant references.

### Macroscopic Simulations

In macroscopic methods, the EG is generally treated as a continuum layer with a limited number of bio-features, and the simulation outcomes are the macroscopic properties of the endothelial dendritic structures. Computational fluid dynamics (CFD; Tu et al., 2019) and the finite element method (FEM; Zienkiewicz et al., 2005) are two commonly used techniques in macroscopic simulations, which originated from the fluid mechanics and solid mechanics communities, respectively. Generally, continuum-based conservation laws and macroscopic physical models for bio-features are solved to obtain flow profile over and/or through the EG layer (Wang, 2007; Lee et al., 2016, 2020), deformation/force of the anchoring cells or membranes (Tarbell and Shi, 2013; Dabagh et al., 2014), order of magnitude of force transmitted via the glycocalyx (Saez et al., 2019).

In numerical modelling, simplifications of the problems under concern are inevitable due to the complexity of EG structures. With a focus on different aspects of the EG features, mechanotransduction can be approached from different perspectives. For example, the endothelial surface is covered by a layer of interweaving, dynamic and brush-like GAG chains. Such a layer can be simplified as an array of side chains or a layer of porous structures. By assuming the EG layer as an organised array of elastic chains, with individual chains having one end fixed to the cell membrane, Secomb et al. (2001) were able to identify the role of EG in transmitting shear stress to the endothelial cell surface and conclude that the EG layer would mediate rapid flow fluctuations and attenuate shear stress. By treating the EG layer as a porous medium, Tarbell and Shi reported that the solid stress of the endothelial cell surface is one to two orders of magnitude higher than the surface fluid stress in the presence of EG layer, suggesting that the interstitial flow shear stress can be sensed by the EG layer (Tarbell and Shi, 2013). As such, the role of EG is to strengthen the mechanical signals. Combining these results, a possible pathway for force transmission via the EG layer is proposed, i.e., flow shear stress → EG → cell membrane, as discussed in Tarbell and Pahakis (2006).

Simplification based on experimental observation is imperative for achieving numerical results of acceptable accuracy in reasonable timeframes. Take geometric periodicity as an example: Researchers (Lee et al., 2020) have set up different periodic geometries for core proteins and GAGs and analysed the pressure gradient (an indicator used to quantify mechanotransduction in the research) differences among different periodic geometries. Results suggest that the periodicity type determines the pressure gradient within the EG layer: hexagonal periodicity of the core proteins leads to greater mechanotransduction, and rectangular periodicity of aggregated GAGs leads to the greatest conversion of pressure gradients to wall tractions. Indeed, a hexagonal periodicity is favoured by previous studies (Weinbaum et al., 2003; Zeng et al., 2013), and conclusion based on the hexagonal periodicity is sensible.

For macroscopic methods, a “complete” model with all the features of the system is definitely beneficial for accurate results from numerical simulations. On the other hand, the computational cost would increase as more details are involved in computational models. Such a complete model is intimately related with access to accurate structural information which in turn relies on the availability (and possibly invention) of cutting-edge microscopic observation equipment.

### Mesoscopic Simulations

The lattice Boltzmann method (LBM) and the dissipative particle dynamics (DPD) are two widely used mesoscopic methodologies in mechnotransduction studies. Both methods are based on the kinetic theory of particle dynamics.

In the framework of LBM, the fluid is modelled as a set of fictitious particles, and such particles interact with each other on a regular discrete lattice mesh through a two-stage “collision and streaming” process. A systematic review of the LBM in flow studies can be found in Chen and Doolen (1998) and Li et al. (2016). In DPD models, the system of interest is simplified to a set of interacting beads, each bead representing a cluster of constitutive molecules or atoms. The governing equation in the DPD method is Newton’s Law of Motion, i.e., *F* = *ma*, where *F* is the force on the bead, *m* is the mass of the bead, and *a* is the acceleration of motion of the bead. The force term comprises three elements: a conservative force, a random force and a dissipative force (Liu et al., 2014). A detailed review of DPD can be found in Espanol and Warren (2017). The particle-based nature of both mesoscale methods facilitates the construction of complex structures at the fluid-structure interface. Thus, recent mesoscale simulation studies are mostly about interpreting the fluid-structure interactions between the blood flow and the complex endothelium surface structures (Yin and Zhang, 2013).

At the mesoscales, more features of the glycocalyx can be included, which enables detailed comparison with experiments. By coupling the LBM with immersed boundary methods, alongside structural solvers, researchers revealed a broad range of behaviours for slender structures, from a single flap in a periodic array, to a small finite array of flaps, and finally to a large finite array (Yin and Zhang, 2013; O’connor et al., 2016; O’connor and Revell, 2019). The findings suggest that the flow instability over the slender array depends on the natural frequencies of the flow and the surface structures (O’connor and Revell, 2019). As a simple extension, one may expect that the shedding or impairment of EG chains would change flow patterns, cause flow instability and further disturb mechanotransduction. The role of EG in regulating flow field was also demonstrated in a recent DPD study (Jiang et al., 2021).

Beyond the fluid-structure interactions between the blood flow and EG layer, mesoscopic methods have been applied to a wide spectrum of haemodynamic problems, like cell suspension and leukocyte adhesion over endothelium (Yan et al., 2019), the glycocalyx–endothelium–erythrocyte interaction in the microcirculation (Pontrelli et al., 2015; Jiang et al., 2021) and the formation of the cell-free layer (Deng et al., 2012).

### Microscopic Simulations

**FIGURE 2:**
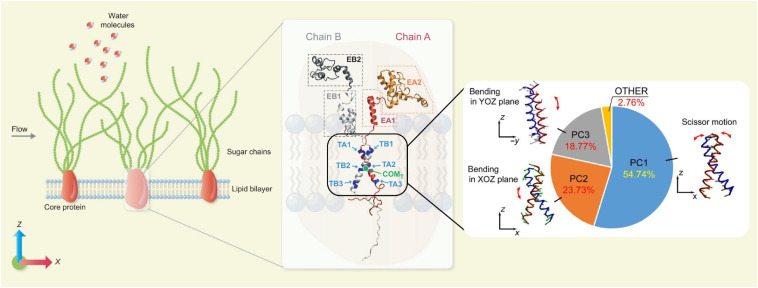
Force transmission mode of Syndecan-4 revealed by MD simulations. **(Left)** An MD model for EG. **(Middle)** Secondary structure of Syndecan-4. **(Right)** Force transmission mode via the transmembrane part of Syndecan-4 (Jiang et al., 2020b; reuse of figure under CC BY 4.0 permission).

Molecular dynamics (MD) is a typical microscopic simulation method that describes how atoms and molecules move and interact during a period of time. The governing equation of MD is also Newton’s Law of Motion as in the DPD method, but a wide range of interatomic and intermolecular forces can be included and often parameterised as force fields. For the theoretical and numerical details of the MD method, the readers are referred to Frenkel and Smit (2002) and Rapaport (2011). When the structural configurations of biomolecules are known and a set of force field parameters are properly chosen, the trajectories of the constitutive atoms can be calculated by MD and the behaviour of the biomolecular system is then fully characterised. In principle, all biophysical problems could be tackled by MD if the structural information at the atomic scale were known. The bottleneck preventing the broad application of MD is its high computational cost, which limits not only the number of observed individual molecules to be included but also the duration of observations (i.e., simulated physical time). Despite such constraints, MD has been increasingly employed in studying dynamics of EG and surrounding molecules, thanks to its unique ability to gain atomic level insight. In the classic vasoregulation signalling problem – *in what manner does glycocalyx transmit force to the cytoskeleton from the flowing blood?*, the glycocalyx was regarded as a rigid body and the force was transmitted by the local torque induced by the movement of the glycocalyx core protein. In recent MD simulations, Jiang et al. (2020b) elucidated the dynamics of Syndecan-4 (a typical type of transmembrane protein of the glycocalyx), and concluded that a *scissor-like* motion of the Syndecan-4 transmembrane dimer, together with its bending, is responsible for the force transmission and the force transmitted into the cytoplasm is of an order 10–100 pN. The MD model and force transmission mode is summarised in Figure 2. The same all-atom model for MD has also been applied to understand how the local lipid membrane around glycocalyx core proteins responds to the flow shear stress (Jiang et al., 2020a). By artificially switching off the charges of the core protein, the contributions of electrostatic and van der Waals interactions to cell membrane deformation induced by flow were quantified for the first time (Jiang et al., 2020a). The capability of isolating specific effects in MD demonstrates an important advantage of *in silico* experiments in exploring the mechanisms of mechanotransduction.

It is worthwhile to mention that fluid-structure interactions between blood flow and EG can also be interrogated by MD. When the current supercomputing platforms are employed, large-scale MD simulations are able to tackle flow problems in the proximity of the EG layer. As reported in recent studies, the blood flow changes the conformation of EG (Cruz-Chu et al., 2014; Pikoula et al., 2018), and the EG spatial configuration in turn modifies the blood flow profile (Jiang et al., 2017, 2018). This is in accordance with the clearly detrimental cycle between the endothelial dysfunction and the degradation of glycocalyx (Zhang et al., 2018).

Together with MD, the Monte Carlo (MC) method, based on the stochastic theory, is also able to capture microscopic features of bio-systems. A recent MC study has demonstrated that the glycocalyx promotes cooperative binding and clustering of adhesion receptors (Xu et al., 2016). The role of glycocalyx in nanocarrier-cell adhesion is also investigated by the MC method (Agrawal and Radhakrishnan, 2007).

## Discussion

In the previous sections, the main features of macroscopic, meoscopic and microscopic simulation methods and their applications in the EG studies are reviewed. Each of these methods (i.e., CFD, FEM; LBM, DPD; MD, and MC) is designed to cover a specific range of length and time scales, and plays a different role in revealing the multiscale multiphysics nature of EG-related phenomena. These methods, however, are not mutually exclusive and can be used in combination. For example, MD, DPD, and LBM have all been used to study the fluid-structure interactions between blood flow and EG, which provide different levels of understanding. MC and LBM have been both used to explore the acceptor-receptor binding, revealing the stochastic and deterministic features, respectively. Moreover, the cell membrane deformation phenomena have been investigated by continuum models and MD, which can give interesting comparisons between macroscopic and microscopic properties of the same process. In short, a hierarchy of numerical simulations have been developed and applied to EG studies, which complement experiments and provide time-dependent and 3D information.

In this mini review, we have focused on the biophysical aspects of mechanotransduction, as the vast majority of numerical studies have been devoted to so far. The biochemical aspects of mechanotransduction, on the other hand, have been rarely studied using numerical simulations, due to the added complexity of biochemical reactions, like the generation of NO (Ignarro, 1989; Bartosch et al., 2017). In principle, the hierarchy of numerical methods can all be developed to reproduce the biochemical processes in mechanotransduction. For example, quantum mechanics/molecular mechanics (QM/MM) computations, which fall under microscopic methods (subatomic scale, to be precise) and advances in probing the electron transfer process, can be applied to find reaction mechanisms of enzymes, like the activation of eNOS (endothelial nitric oxide synthase) as reviewed in Bignon et al. (2019). Also, reactive force field MD (Senftle et al., 2016) simulations, which uses QM-trained force fields to mimic bond breaking and formation and have been extensively proved to be effective in capturing reaction pathways, can be applied to work out the chain reactions after the release of Ca^2+^ initiated by mechanotransduction.

Although mechanotransduction has been investigated using a multitude of methods, in most case, these methods are applied individually or sequentially. A truly multiscale numerical method requires the dynamic coupling of different simulation methods in one simulation (Fedosov and Karniadakis, 2009). For example, a coupling between DPD and MD via a special multi-scale interface server (Wang et al., 2019) could solve the fluid problem and force transmission simultaneously. The challenge, however, is to find a suitable mapping between high- and low-resolution simulations in both spatial and temporal senses. Despite such inherent difficulties, dynamic coupling of simulations is expected to play an ever increasing role in studying multiscale phenomena such as mechanotranduction, as more powerful and cheaper computing platforms become as available.

Finally, we anticipate increasingly coupled use of artificial intelligence and numerical simulations in the coming years. Already, machine learning techniques have been used to empower MD to simulate a molecular system of 100 million atoms with *ab initio* accuracy (Jia et al., 2020), which is unprecedented. Together with the advent of exascale supercomputers, the integration of artificial intelligence and big data strategies into numerical simulations will revolutionise the way to conduct *in silico* experiments, leading to better understanding of the detailed mechanisms of mechanotransduction. Another significant development based on numerical simulations is the construction of a digital twin for a bio-system such as an EG layer, which will enable real-time diagnosis of diseases and, better still, instant repair of diseased components.

## Author Contributions

XJ drafted the manuscript. KL and YV offered the direction, proposed ideas, and revised the manuscript. All authors contributed to the article and approved the submitted version.

## Conflict of Interest

The authors declare that the research was conducted in the absence of any commercial or financial relationships that could be construed as a potential conflict of interest.

## Publisher’s Note

All claims expressed in this article are solely those of the authors and do not necessarily represent those of their affiliated organizations, or those of the publisher, the editors and the reviewers. Any product that may be evaluated in this article, or claim that may be made by its manufacturer, is not guaranteed or endorsed by the publisher.
